# Tracking Psychodynamic Foci: Trajectories Through the Therapeutic Process

**DOI:** 10.3389/fpsyg.2022.786240

**Published:** 2022-06-06

**Authors:** Paula Dagnino, Ana Calderón

**Affiliations:** ^1^Facultad de Psicología, Universidad San Sebastián, Santiago, Chile; ^2^Millennium Institute for the Study of Personality and Depression, Santiago, Chile; ^3^Escuela de Ciencias Sociales y Humanidades, Carrera de Psicología, Universidad Gabriela Mistral, Santiago, Chile

**Keywords:** brief psychodynamic psychotherapy, conflict focus, relational focus, personality functioning focus, patients’ subjective change

## Abstract

Brief psychodynamic psychotherapy has gained importance in current clinical practice. To achieve brevity, a focus must be established and worked through. Different conceptualizations have emphasized the relational patterns and/or conflict foci as central but adopting a mono-schematic approach. However, patients come to treatment with more than one issue that must be addressed. Thus, another focus must be included because of its relevance, i.e., personality functioning. The aims of this study were to identify the presence and depth level of three foci (relational pattern, conflict, and personality functioning) in episodes of change throughout the process, and to evaluate the relationship of each focus with the complexity of patients’ change. Initial OPD foci and the presence and depth of each were evaluated in 13 successful brief psychodynamic therapies. Change episodes of those therapies were analyzed as well. Results showed differences between foci in the initial phase with a higher presence of conflict focus. Throughout the process, only the presence and level of personality functioning improved. Also, complexity of patients’ change was related to conflict focus, specifically on the emergence of competence feelings. The results provide evidence and enrich process research of brief psychodynamic therapies.

## Introduction

In recent decades, brief psychodynamic psychotherapy has gained importance in current clinical practice (e.g., Guthrie et al., 1999) especially in public mental health services where the high demand they are exposed to impel them to provide shorter treatments. However, most of these are brief therapies because of external factors rather than by design (Budman and Gurman, 1988). Brief psychodynamic psychotherapy is not just a shorter version of a longer psychotherapy process (Cummings, 1987), and therefore therapists must be trained specifically in this type of psychotherapy (Levenson et al., 1995).

Compared to long-term psychodynamic psychotherapy (or even psychoanalysis), brief psychodynamic psychotherapy is adjustable to a broad range of patients, it impels therapists to be active, to make a rapid assessment, to quickly establish therapeutic alliance and to be oriented to the here and now. It also implies that therapists must propose a treatment contract with therapeutic goals that could be achieved in the limited time they will have (McWilliams, 1999; Ursano et al., 2004; Levy et al., 2012).

Brief psychodynamic psychotherapy has shown to be effective (Koss and Shiang, 1993; Piper et al., 1998; Hoglend, 2003; Leichsenring et al., 2004; Hilsenroth, 2007; Cuijpers, 2017). However, for setting up a brief psychodynamic therapy one or more foci must be established and worked through, in comparison to more comprehensive goals built in long-term treatments (Budman and Gurman, 1988). This is because the focus allows the consolidation of the material and makes it possible to shorten the process (Balint et al., 1972; Scaturo, 2002).

Therefore, the psychodynamic focus can be considered the center around which brief psychotherapy is organized (DeLaCour, 1986; De la Cerda and Dagnino, 2021). The focus can be established upon the material provided by the patient and the therapist’s ability to perceive and understand it (Thomä and Kächele, 1987; Dagnino, 2012). Its formulation and presentation are clearly intended to match the felt experience of the patient (Smith, 2006), and it serves to protect both therapist and patient from becoming overwhelmed by all the clinical material (Thomä and Kächele, 1987; Mander, 2002).

The therapist from an active, inquiring position must establish the focus early in the process and must work on it constantly. To do so, they must have a selective inattention, i.e., not to pay attention to material that is not related to the established focus. This is different in a psychodynamic therapy of unlimited time where the therapist is in a less active listening attitude and has free-floating attention to all the material brought by the patient (Mander, 2002; Messer and Warren, 1995; Summers and Barber, 2010).

The work on the psychodynamic focus is so pivotal that it is considered a change mechanism itself (Poch and Maestre, 1994) because it gives shape and form to the patient’s material (Dagnino, 2012) and is a container of the inarticulated felt experience (Malan, 1976; Marziali, 1984; Crits-Christoph et al., 1988; Messer and Warren, 1995; Safran and Muran, 2000; Smith, 2006).

### Definition of Focus

The conceptualization and operationalization of the psychodynamic focus have been addressed by many authors, some of whom have developed operationalizations and even therapy models (e.g., Malan, 1963; Luborsky, 1984; Fosha, 2000; McCullough and Magill, 2009; Abbass et al., 2017). All of them emphasized the relevance of identifying the psychodynamic focus early on and working on it during the psychodynamic process. Each of these focus definitions have emphasis on different aspects. For example, one of the first in conceptualize the therapeutic focus, Malan (1976), described “focality” (p. 11) as an attempt by the therapist to tackle the patient’s “basic neurotic conflict” (p. 13), which was part of what he called the triangle of conflict. Strupp and Binder (1984) developed a conceptualization of the focus that represented a map of the main areas of the patient’s dysfunctional mental functioning and maladaptive relationship behavior called cyclical maladaptative pattern (Schacht et al., 1984). For Luborsky (1984), the focus had to do with a patient’s interpersonal relationships with important people in his or her life and the therapist (i.e., core conflictual relationship theme).

Despite the diversity of definitions and models, the similarities these authors present can be grouped into two dimensions. The first could be called “type of focus”, which points out to the *theme* of the focus. In this dimension two categories arise: those that emphasize interpersonal patterns (e.g., Luborsky, 1977); and those that refer to specific intra-psychic conflicts (e.g., Sifneos, 1972; Wallerstein, 1989). The first highlights the dysfunctional relationships as the fundamental factor in the development and continuity of disorders (e.g., Benjamin, 1974; Luborsky, 1977; Strupp and Binder, 1984). The second category (intra-psychic conflict) focuses on unconscious conflicts, clashing forces and tensions as the generators of the patient’s disorder (e.g., Sifneos, 1979; Perry et al., 1989; Wallerstein, 1989; McCullogh Vaillant, 1997).

The second dimension alludes to the *composition* of the focus: mono-schematic or multi-schematic (Barber and Crits-Christoph, 1993; Dagnino, 2012). This has to do with whether or not the authors consider the need to establish one or more foci at the beginning of the process. Mono-schematic has been the most prevalent conceptualization so far, but clinical wisdom and some research on the subject have shown that the reality implies a more complex approach, i.e., a multi-schematic approach (Crits-Christoph et al., 2005). In fact, this can be found in Balint’s early work (1972), when he proposed the existence of reference points to elicit therapeutic change. Considering the presence of multiple foci that are mutually agreed upon at the beginning of therapy and constantly borne in mind thereafter reduces the danger of the therapist imposing only one aspect of the dynamic on the patient (Thomä and Kächele, 1987). Multiple foci can change over time, enabling a sense of narrative cohesion that weaves together apparently unrelated sessions and helping organize the therapeutic experience (Coren, 2001; Dagnino, 2012).

### Dynamic Diagnosis – Focalization

A psychodynamic formulation is essential for the foci identification. Among the systems that allow us to do a thorough assessment it could be found the *Psychodynamic Diagnostic Manual* (PDM Task Force, 2006), the *Shedler Westen Assessment Procedure* (SWAP; Shedler and Westen, 2007), the *Karolinska Psychodynamic Profile* (KAPP, Weinryb and Rössel, 1991), and the *Operationalized Dynamic Diagnosis* (OPD; OPD Taskforce, 2001, 2008). The latter is used in this study since it has had a high acceptance among psychotherapists in the national context; there has been an increase of psychotherapists in the system’s training courses and has been progressively more used in mental health care centers. The OPD system allows to identify multiple foci that could be worked during the psychotherapeutic process. For OPD the foci are specific areas that are significant to the patient’s psychodynamics (Grande et al., 2004), and support the patient’s psychic or psychosomatic symptoms. This emphasizes the need for a distinctive comprehension of each patient and to consider change processes that are unique to each dyad (Ablon and Jones, 2005).

### Focus Areas in Operationalized Dynamic Diagnosis

Operationalized dynamic diagnosis can identify as foci three main areas. Two of these were mentioned above by the authors who conceptualize the focus: dysfunctional interpersonal pattern and internal conflictual configuration. A third, new area is personality functioning which is also called *structural vulnerabilities* (in this article, these terms will be used interchangeably).

Regarding the *dysfunctional relational pattern*, many studies have shown that they are a major reason why patients seek help (Strupp and Binder, 1991). They are also fundamental to psychotherapy because a change in the patient’s mental representations result in a more flexible way of dealing with others, in turn providing more satisfaction with their interpersonal relationships (Cierpka et al., 2007; Gross et al., 2007). The OPD evaluates the relational pattern as a circular matrix of interaction and stands apart from other instruments because of its comprehensiveness, intermediate complexity, and inclusion of the interviewer’s subjective experience in the identification of the interactional pattern (Dagnino, 2012).

In the *internal conflictual configuration*, the definition of conflict is not based on the traditional psychoanalytic conception but assumes that human behavior is constantly influenced by desires, thoughts, and unconscious representations (OPD, 2008). It refers to unconscious coalitions between basal motivational groups, e.g., the basic desire for care versus autarchy. It assumes that the internal pressure and opposite motives, maintained over a long period of time, lead to an elevated internal state of tension (Dagnino, 2012).

In relation to *personality functioning*, clinicians and researchers are moving away from the diagnosis of personality disorders to evaluate how personality functions (*Diagnostic and Statistical Manual of Mental Disorders* (5th ed. [DSM–5]; American Psychiatric Association, 2013) Section III Alternative Model for Personality Disorders (AMPD). When delivering psychodynamic psychotherapy, it is crucial to focalize on relevant topics or aspects of a patient’s functioning (Diener et al., 2007; Luyten et al., 2012; Drapeau et al., 2018). In OPD, personality functioning includes several psychoanalytic traditions such as ego-psychology (Bellak and Hurvich, 1969), self-psychology (regulation of self-esteem, self-reflection, identity), and internalized object relations (Kernberg, 1975).

The relational pattern, conflict, and personality functioning foci are interrelated. In fact, patients begin therapy troubled by more than one issue, requiring a multi-focal approach (Barber and Crits-Christoph, 1993; Dagnino, 2012). The interaction between foci can follow two paths. First, the relational pattern represents a surface where conflict can show up, and the quality of this engagement or coping is eventually directed to the patient’s functional capacities. The other path considers that personality functioning (co)determines the quality and character of the other foci, i.e., the extent of the personality vulnerabilities influences the difficulties the patient has acquired in his or her development (Rudolf et al., 2002; Dagnino, 2012).

No matter which path is followed, conflict and personality functioning relate like content and form (Mentzos, 1991); conflict refers to the “why” and personality functioning to the “how” of a disorder. They may be expressed through dysfunctional relational patterns as the manifestation of conscious or unconscious aspects of the psyche (OPD, 2008). In fact, relational patterns could be considered as a byproduct of the personality functioning and internal conflicts (Grande, 2007). Therefore, the work on the relational pattern may represent an indirect work on conflict or personality functioning.

### Foci in the Psychodynamic Process

The foci can also be understood as nodal points in a network of dynamic interrelations throughout the process (Dagnino, 2012). Some authors have suggested that a particular focus can dominate the therapy at one point in time and may subsequently be replaced by another (Balint et al., 1972; Thomä and Kächele, 1985; Stauss and Fritzsche, 2008; Summers and Barber, 2010). However, after identifying the foci no study has sought to look at how they are present throughout the psychotherapeutic process. This is the main aim of this study. As the psychotherapeutic work on foci is considered a mechanism of change (e.g., Messer and Warren, 1995) it is not only researching the psychotherapeutic process of brief psychodynamic therapy but also the mechanisms of change that allow patients to get better.

The most (clinically) useful way to research the psychotherapeutic process is through its segmentation into minor units of analysis as it is through this fine-grained analysis that the essential nature of the mechanisms leading to patients’ change can be understood (Rice and Greenberg, 1984). There are many labels for these segments, such as critical events (Fitzpatrick and Chamodraka, 2007), significant events (Elliott, 1989; Elliott and Shapiro, 1992), or change episodes (Krause et al., 2007). The latter designation is used in this study since it identifies fragments of sessions in which there is an intensification of the process of change that culminates in a specific moment of change (De la Cerda and Dagnino, 2021). From this viewpoint, change is in the modification of the subjective interpretation and explanation of the image of oneself and the world, which leads to new subjective theories (Groeben et al., 1988). These changes evolve throughout the therapeutic process, resembling a saw tooth pattern (Caro Gabalda, 2006), but with greater change complexity in the process of successful cases (Krause et al., 2007).

It is expected that the work on each focus can appear through change episodes at different depth levels and that this work will relate with the level of change complexity accomplished by the patient. This, in turn, will allow the patient to integrate these changes into his or her everyday life (Malan, 1976; Marziali, 1984; Crits-Christoph et al., 1988; Messer and Warren, 1995; Safran and Muran, 2000).

Considering the above, the aims of this study were to (1) identify the presence and depth level of each focus at the beginning, middle, and final phases of the psychotherapeutic process, (2) evaluate how each focus changes during the psychotherapeutic process, and (3) evaluate the relationship between foci and the level of patients’ change (level of hierarchy). It is expected that the focus on personality functioning will be more present in the initial phase since it supports the work on the other foci. When looking at the trajectories of each focus during the process, it is expected that the relational pattern focus will be stable and the conflict focus will increase its presence throughout the therapies, especially in the middle part of the process. It is also expected that the personality functioning focus will decrease in favor of conflict focus. Finally, it is expected that patients’ subjective change will be more complex when the presence and level of conflict focus is higher.

## Materials and Methods

### Design and Participants

The study used a *multiple single subject* design with mix analysis. The sample comprised 13 psychotherapeutic processes of brief psychodynamic psychotherapy, which were selected from a database with 25 individual psychotherapeutic records from different theoretical orientations, collated during FONDECYT projects 1030482 and 1060768. All psychotherapies were conducted in Chile and had a maximum of 25 sessions (mean = 18), with a weekly frequency, in a face-to-face modality.

The inclusion criteria for the selection of the 13 psychotherapeutic processes were two: (1) psychodynamic therapeutic orientation and (2) successful outcome using the Outcome Questionnaire (OQ-45.2, Lambert et al., 1996). Specifically, a total reliable change index (ICC) higher than 17 points (De la Parra and Von Bergen, 2001) was considered successful, since it is the amount of change considered to be reliable beyond statistical error.

Change episodes were the unit of analysis. A total of 208 change episodes were identified through the GchI (Krause et al., 2007, see procedure). They were extracted from 246 psychotherapy sessions. The number of episodes of change within therapeutic processes ranged from 5 to 46, with a mean of 16 episodes (*SD* = 11.35).

Patients were mostly single women (Table 1) who consulted at various mental-health outpatient care centers. Reasons for consultation varied, although all of them presented mainly depressive symptoms (according to the clinicians’ diagnosis).

**TABLE 1:** Patients’ descriptive statistics (*N* = 13).

Patients	*N* or *Mean (SD)*
**Gender**	
Women	12
Age	39 (13)
**Marital status**	
Married	4
Divorced	2
Single	6
Widow	1
**Level of education**	
Unfinished school	1
Technical	2
Professional	10
Initial personality functioning (OPD-2)	1.8 (0.4)

There were eleven therapists – six women and seven men – with a mean age of 42 years (*SD* = 5). They had at least a master’s degree, psychoanalytic training or training in brief psychodynamic psychotherapy. Two of them – one woman and one man – did two psychotherapeutic processes, the rest conducted only one. Psychotherapists had a mean of 22 years of experience (*SD* = 3). None of them had OPD training or knew about the system when conducting the psychotherapies.

The initial foci, idiosyncratic for each patient, were identified using the OPD-2 manual (see procedure).

### Instruments

#### Outcome Questionnaire (OQ-45.2)

This 45-item self-report questionnaire was developed by Lambert et al. (1996). It measures psychotherapeutic outcomes across three areas: (a) symptomatology (e.g., “I get tired quickly”), (b) interpersonal relationships (e.g., “I get along well with others”), and (c) social role (e.g., “I feel pressured/stressed at work/school”). Each item is scored from 1 (never) to 4 (almost always) on a Likert scale. The higher the score, the greater the psychological discomfort. The OQ-45.2 was validated in Chile (De la Parra and Von Bergen, 2001), finding a clinical cut-off point of 73 and a reliable change index of 17 points. The test/re-test reliability was 0.90 (for the total OQ) and Cronbach’s alpha was 0.91, for both the clinical and non-clinical sample.

#### Personality Functioning (OPD-SQ)

Personality functioning was assessed at baseline through the self-report OPD structure questionnaire (OPD-SQ, Ehrenthal et al., 2012). This 95-question instrument assesses eight personality dimensions. Each item is scored on a five-point Likert scale, from “I do not agree” to “I totally agree.” Higher scores imply lower levels of personality functioning. The mean of all the scales is an indicator of overall personality performance. Reliability studies in Chile have shown that Cronbach’s internal consistency ranged between 0.92 and 0.68 for the full sample, between 0.91 and 0.67 for the clinical sample, and between 0.87 and 0.48 for the healthy control sample (Lorenzini et al., 2021).

#### Operationalized Psychodynamic Diagnosis

The foci identification was made through the scoring procedures of the OPD manual (see OPD Taskforce, 2008). In the interview(s) the following current complaints and problems were explored: information of early family life, relationship experiences with parents and significant others, personal development during later years, friendships and romantic relationships, education, and work history. The three possible foci are: relational pattern, conflict, and personality functioning. The manual provides detailed examples and a checklist to facilitate the rating of each focus.

#### Foci Presence and Depth Scale

The Foci Presence and Depth Scale (FPDS) was developed by Dagnino and de la Parra (2010) and measures both the degree of presence and depth level of a focus. This is achieved by analyzing a verbal interaction between a patient and a therapist. The FPDS allows to identify foci in segments of psychotherapy sessions. It requires a formulated OPD initial focus which is then contrasted with the video-recorded or transcribed sessions. With the transcription of therapy segments, two raters (in this study, psychotherapists trained in OPD with ten or more years of clinical experience) identified the level of presence of the focus that was being worked upon. When no OPD focus could be identified, raters had to describe the theme of the segment with a score of 0 (absence of foci). When a focus could be identified the following scores could be given: 1 (vague reference), 2 (knowledge and exploration of focus), or 3 (work on focus). A single intra-class correlation coefficient (ICC, Shrout and Fleiss, 1979) was used for reliability since the variables were continuous. A previous study has shown an ICC range between 0.57 and 0.80 for relationship pattern; an ICC range between 0.75 and 0.91 for conflict focus, and an ICC range between 0.72 and 0.82 for personality functioning (Dagnino, 2012). According to Fleiss (1981), these can be considered as fair to excellent for the relationship focus and as excellent for the other two foci.

#### Generic Change Indicators

Moments of change were identified through a list of nineteen generic change indicators that go from the least to the most complex [Generic Change Indicators (GCIs), Krause, 2005; Krause et al., 2007; see Annex 2]. The criteria they had to fulfill in order to be considered episodes of change were the following: (a) the moment of change has to coincide with at least one GCIs; (b) there has to be consistency, which refers to the concordance between the verbal and non-verbal cues for the verbally expressed change of each patient, and the fact that subsequent moments of the session or therapy should not contradict it; and (c) the event had to be new on the process. Even though the 19 GCIs may appear more than once during a particular therapy, the specific theme to which this change refers should be coded just once.

### Procedure

*First phase: change episode identification.* To identify change episodes two independent trained raters observed *in situ* the psychotherapy and coded all the change episodes they could identify. When the session ended, raters compared their codes. For the episodes in which they did not agree, the research team discussed until consensus was reached (inter-subjective validation) (Hill et al., 1997).

**FIGURE 1:**
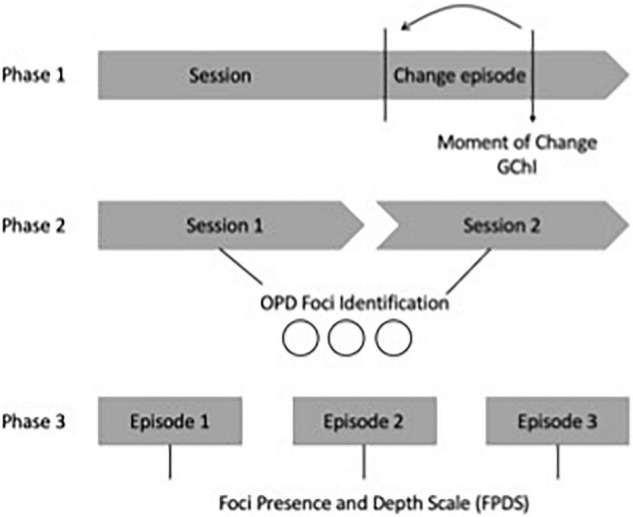
Phases of the procedure.

As shown in Figure 1, *change* episodes are first identified by singling out a *moment* of change (which constitutes the end of the episode). Then, this is worked backward; to identify when the interaction related to the theme started the rater revise the preceding interactions until they found the starting point [for more information regarding this procedure please refer to Krause et al. (2007)].

Raters on the second and third phases were therapists formally trained in OPD (OPD Taskforce, 2008), who underwent 100 h of theoretical and practical training and applied the method to clinical interviews.

*Second phase: foci identification:* two raters identified the OPD foci (dysfunctional relational pattern, main conflict, and main vulnerability of personality functioning) from the videos and transcripts of the first two interviews of the 13 therapies of the sample.

*Third phase: foci presence and depth level:* The first author received the identified foci for each patient. Then she prepared a document that included clinical vignettes and descriptions of how these foci could be observed in the material. This document, the videos and transcripts were provided to a second group of raters. They received the transcripts of each change episode randomly to avoid any bias. Hence, they did not know which session of the process the transcripts corresponded to Dagnino (2012).

### Ethical Approval

All 13 processes had signed an informed consent that authorized the use of such records for research purposes, not limited to the project in which they were conducted. Authorization for the use of these data for the present study was granted by the Ethics Committee from Pontificia Universidad Católica de Chile and Universidad Alberto Hurtado, following the declaration of Helsinki (64th WMA General Assembly, Fortaleza, Brazil, October 2013).

### Data Analysis

When analyzing the therapeutic process, psychotherapeutic phases were constructed considering the number of sessions of therapy divided by three (i.e., beginning, middle, and final). For example, for a therapy that had 13 sessions, 5 were labeled “initial,” 4 were labeled “middle,” and 4 were labeled “final.”

Focus intensity was a variable constructed based on each focus presence and depth. To construct this new variable, it was first identified which focus was present and then the score for the depth of that focus was copied into the new variable. So, when the dysfunctional relational pattern (Focus 2) was present, the depth of the dysfunctional relational pattern was copied to the new variable; when the conflict focus (Focus 3) was present, the depth of the conflict focus was copied; and when the personality functioning focus (Focus 4) was present, the depth of the personality was copied. In the first and second objectives, the analyses were conducted considering the focus intensity as a continuous variable. For the third objective the focus intensity was treated as dichotomic variables: low presence and depth (scores from 0 to below 2) and high presence and depth (scores from 2 to the highest).

Complexity of change was considered a continuous variable following the GChI instrument. This is a continuous variable with 19 change indicators hierarchically ordered from least to most complex.

Two generalized estimating equations (GEE) were used, as data were nested within therapeutic processes. The two GEEs had as the dependent variable the focus intensity. In the first GEE, the dependent variable was the focus intensity measured as a continuous variable, and the within-subject effects were the phase to which the session belonged to and the type of focus. Pairwise comparisons were adjusted for multiple comparisons using Bonferroni. This analysis informed the first and second objectives.

The second GEE had as a dependent variable the complexity of change measured as a continuous variable and the within-subject effect were the focus intensity (as categories) and the type of focus. Pairwise comparisons were also adjusted for multiple comparisons using Bonferroni. This analysis informed the third objective.

## Results

### Foci Presence and Depth Throughout the Therapeutic Process

The focus intensity in the three phases and the type of focus were examined. Results showed that the model was significant [χ^2^ (8 *df*) = 42.59, *p* ≤ 0.001, Cramer’s V = 0.26]. To explore where the differences were, pairwise comparisons using Fisher’s Least Significant Differences were conducted.

**FIGURE 2:**
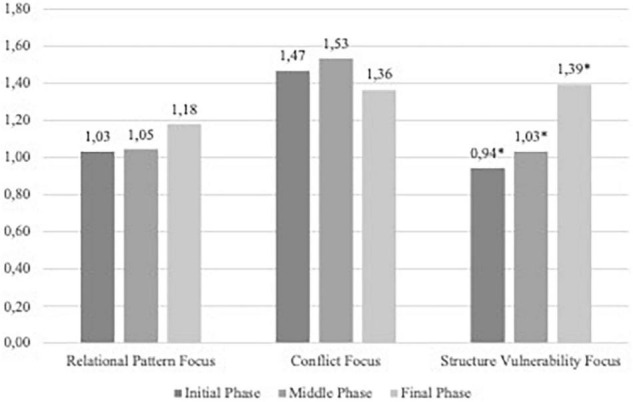
Foci intensity in the three phases and the type of foci.

When the phases were analyzed, significant differences were found in the focus intensity in the initial phase between conflict and structural vulnerability (Mean difference: 0.525, *p* = 0.04), as Figure 2 shows.

When each focus was analyzed, significant differences were found in the focus intensity for structural vulnerabilities between the initial and final phase (Mean difference = −0.449, *p* = 0.04), and between the middle and the final phase (Mean difference = −359, *p* = 0.036). No differences were found in this focus when comparing the initial and middle phase, even though an increased tendency can be observed. The other two foci (relational pattern and conflict) did not show any difference throughout the process.

### Foci Presence and Depth and Subjective Change

**FIGURE 3:**
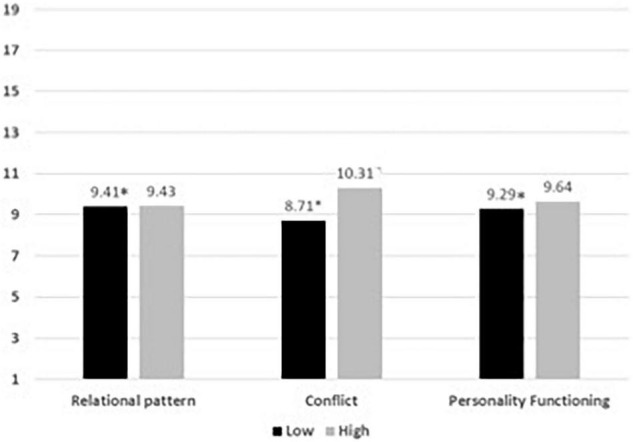
Average of the level of complexity of patients’ change and the high or low presence of each focus on the whole process. ^∗^*p* < 0.05.

The complexity of the patients’ change was predicted by the focus intensity (as categories) and the type of focus. Results showed that the model was significant [χ^2^ (5 *df*) = 18.33, *p* = 0.003, Cramer’s V = 0.17, see Figure 3].

When the focus intensity was analyzed, significant differences were found in the complexity of patients’ change in low intensity between Relational Pattern and Conflict (Mean difference = 0.268, *p* = 0.047), and between Relational Pattern and Structure Vulnerability (Mean difference = 0.499, *p* = 0.01). No other significant difference was found.

Finally, from the figure we can see how the complexity of patient change occurs between levels 8 and 10.

## Discussion and Conclusion

The focus is a central and defining aspect of brief psychodynamic psychotherapeutic work in the private and public clinical practice. This study searched for the presence and depth of three foci, from a multi-schematic perspective: relational pattern, conflict, and personality functioning. This was conducted by identifying each idiosyncratic focus in episodes of change throughout 13 brief successful psychodynamic therapies.

When looking at each phase, the results showed significant differences only in the initial phase, where the conflict focus had greater presence and depth than the personality functioning focus. This analysis presented almost a medium effect size, which represents a medium association. Work on patients’ conflict is closely related to classic psychoanalysis and considered essential for any treatment (Smith, 2003). However, as these were brief psychodynamic psychotherapies, it was not expected to find at the beginning of the therapies significant work on conflicts but rather on personality functioning. This hypothesis was made because in brief psychodynamic therapies there may be the need to work first in the vulnerabilities in personality functioning to then allow the emergence of other focus. In fact, conflict and personality functioning represent polarities in psychic complementarity (Mentzos, 1991). Maybe the predominance of work on conflict in these psychotherapies is related to the fact that all patients in this sample showed medium integration in personality functioning at baseline. This means that there were no major impairments, and therefore the work on personality function was less necessary than the work on conflict.

In addition, results showed that conflict focus tended to increase its presence in the middle phase of the process. The need to work on conflict has been a long tradition in psychoanalysis as a sign of success, especially when patients show insight in these themes (Gabbard, 2004). The work on conflict can be experienced as painful and with an intense emotional correlate. In fact, some authors (Strong and Claiborn, 1982; Kiesler, 1983; Tracey, 1986) mention that the role of successful therapists is to put systematic pressure on relevant patients’ issues (which can be understood as conflict), and this pressure should be gentle during the early stages of the process in order to create a solid and stable working alliance, it must be intense in the middle phase and soft again at the end, when patients have changed. This is precisely what can be observed in the evolution of this focus.

Finally, the work on the personality functioning focus was the only one that showed significant differences as it showed an increment in its presence and depth during the whole psychotherapeutic processes (from beginning to end). In the tradition of psychodynamic focal psychotherapy, many authors refer to the work on personality functioning as the essential work conducted on conflicts between different parts of the psychic structure or on the interpersonal functioning. In fact, Messer and Warren (1995) emphasized that the work on personality functioning excludes the possibility of applying brief psychodynamic psychotherapy, and that they probably correspond to so-called “difficult” patients, which does not fulfill the selection criteria for this type of psychotherapy. What became evident in this study was that the focus on the vulnerabilities of personality functioning is something that psychotherapists do even when they are trained in the psychodynamic orientation. It is therefore necessary to consider that this aspect should be taught in therapists’ training.

As was previously mentioned, it was expected that personality functioning focus would have a higher presence in the initial phase of the process, but the results showed that the work on it increased significantly toward the end of the process. This result may have to do with the need of the therapist to increase self-autonomy through the work on specific functioning impairments; and it may have to do with the brevity of focal psychotherapy and therefore the need to prepare the patient for daily life obstacles thereafter. If the expressive–supportive continuum (Rockland, 1989; Luborsky and Mark, 1991; Gabbard, 1994) is considered part of the work, it can be assumed that in the final phase more supportive interventions will be present, with the aim of decreasing vulnerabilities in personality functioning.

All of the results mentioned above about which foci is more present in each phase and how each focus develops through the process, are innovative and are an initial approach to comprehend the work on psychodynamic brief psychotherapy. The results are also relevant to evaluate the presence and level of focus in relation to the complexity of patients’ subjective change. In general, results showed that the levels of change mostly had to do with an increase in permeability toward new understandings and oscillated between the “discovery of new aspects of the self” (GChI 8), “manifestations of new behaviors and emotions” (GChI 9), and the “appearance of feelings of competence “(GChI 10). Change develops in psychodynamic therapies through facilitating self-awareness and insight (e.g., Barber, 2009; Eppel, 2018). The results presented are in line with this, since it is expected that patients will re-experience old feelings and work through the meanings of some events, which may lead to the discovery of new aspects of the self, and therefore the emergence of new behaviors and emotions.

Specifically, looking at how each focus relates to patients’ subjective change, the only focus that showed significance was the presence and level of the conflict focus. The presence of this focus was shown to have a significant relation with higher levels of subjective patients’ change. This result shows us that the work on intra-psychic conflicts leads to the emergence of feelings of competence in the patient. These feelings may be because, as the old feelings and perceptions re-enter consciousness, patients’ natural problem-solving capacities emerge. However, it is also important to keep in mind that the effect size of this analysis was small; hence, although significant it might not be very meaningful.

In summary, it was found that when comparing the focus in each phase of the process, the focus on conflict is worked more and in greater depth in the initial stage of the process, as opposed to the focus on personality functioning. In addition, it was found that the work on personality functioning increases in presence and depth during the psychotherapeutic process, a tendency of stability is observed in the relational focus, and a tendency to increase in the middle phase could be seen in the conflict focus. Finally, in relation to the changes produced by the patient, they increase in complexity, and only the presence and level of conflict focus had the greatest relationship with higher indicators of change.

### Contributions of This Study

This study provided the basis for generating scientific knowledge of brief psychodynamic psychotherapy. Particularly by doing so through systematic observation and evaluation repeated over time (Chassan, 1979). This type of psychotherapy is and will continue to be a relevant approach especially in institutional work.

An important contribution of this study was the development of the Foci Presence Scale and Depth (Dagnino and de la Parra, 2010). As Grande et al. (2004) argue, there is a need for development of an observational instrument of psychodynamic processes on the aspects worked through. This instrument can be used for training and practice on clinical settings since it encourages to observe the presence of foci in relevant segments. Therefore, practitioners gain a “royal road” to the way psychotherapy works with patients’ problems (Lepper, 2009). Greenson (1967) said that if the therapist knows what to say and when to say it, the interaction between patient and therapist points out to success. Formulating the interventions with knowledge has great impact on a helpful relationship.

### Limitations and Future Research

One of the main limitations of this study is that the sample was composed of patients who showed medium integration of personality functioning. This means that, even though there were vulnerabilities, these were moderate. In further research it would be interesting to see how foci, especially the personality functioning focus, evolve in patients with more baseline impairments (OPD Taskforce, 2008).

Also, as the aim of the study was to study successful psychotherapies and significant segments with change episodes, no comparison with unsuccessful psychotherapies or segments where no subjective change appears (e.g., stuck episodes) were conducted. This limits the conclusions that could be reached because as there is no comparison group, we cannot be certain that what we observed here would not be present in unsuccessful psychotherapies as well.

For future research it would be interesting to look at the association between the presence and level of foci with other elements of change, for example, the course of the alliance between patient and therapist or the therapist’s identification of foci, session by session.

The results of this study reveal the importance of continuing to study therapeutic foci and to do it from a process view. Focalization and interventions on foci are central aspects when developing effective psychotherapeutic processes. This line of research will not only provide knowledge about foci and brief psychodynamic psychotherapy as an effective treatment, but will also help to develop practical orientations for clinical practice.

## Data Availability Statement

The datasets presented in this article are not readily available because of confidentiality reasons. Requests to access the datasets should be directed to PD, pauladagnino@gmail.com.

## Ethics Statement

The studies involving human participants were reviewed and approved by Ethics Committee from Pontificia Universidad Católica de Chile and Universidad Alberto Hurtado, following the declaration of Helsinki (64th WMA General Assembly, Fortaleza, Brazil, October 2013). The patients/participants provided their written informed consent to participate in this study. Written informed consent was obtained from the individual(s) for the publication of any potentially identifiable images or data included in this article.

## Author Contributions

PD contributed to the conception, design, assessment of the study, and wrote the first draft of the manuscript. AC performed the statistical analysis. PD and AC contributed to the manuscript revision, read, and approved the submitted version.

## Conflict of Interest

The authors declare that the research was conducted in the absence of any commercial or financial relationships that could be construed as a potential conflict of interest.

## Publisher’s Note

All claims expressed in this article are solely those of the authors and do not necessarily represent those of their affiliated organizations, or those of the publisher, the editors and the reviewers. Any product that may be evaluated in this article, or claim that may be made by its manufacturer, is not guaranteed or endorsed by the publisher.
