# Supplementary material for: Tracking Psychodynamic Foci: Trajectories Through the Therapeutic Process
Source: Front Psychol. 2022 Jun 6;13:786240. doi: 10.3389/fpsyg.2022.786240 (PMC9207400; doi:10.3389/fpsyg.2022.786240)
Supplement: Supplementary file 1 [file Table_1.DOCX]

**Annex 1**

### Foci Presence and Depth Scale (FPDS; Dagnino, P. & de la Parra, G., 2010)

*Instructions*: The evaluator must base his/her observation on the verbal interaction between therapist and patient during the segment of psychotherapy and must codify the level of presence of foci in each segment, scoring the type of focus that the participants refer to. For this, the rater must consider the focus that has been established for the patient in particular. It is worth pointing out that, in one segment, several foci can be worked, but at different levels of presence and depth, so it is necessary to establish it for each of them (scoring the number of foci in the boxes on the right; for example, on personality functioning there may be three established foci for the patient, and each of them will have a number that must be written on the box). In the case of Level 0, it can happen that patient and therapist are working on a topic that does not belong to the foci identified early on for that patient, so if this level is scored, a small description of the talked-about theme must be written.

Rater: ____ ____ (First letter of mothers’ and fathers’ name rater)

Segment:____

Therapy:___

|  |  | Foci | | |
| --- | --- | --- | --- | --- |
|  |  | **Relational Pattern** | **Conflict** | **Personality Functioning** |
| Level 0  Absence of work on the focus | Patient and therapist do not refer to OPD focus.  If the rater perceives that the focus is being acted (and not explicitly formulated) you must consider this level. |  |  |  |
| Level 1  Vague reference to focus | Any of the participants refers vaguely the focus, that is to say that the rater has to be more inferential or go to higher levels of abstraction to deduce the focus, since this is not so evident.  For example, in the case of conflict focus the allusive theme is seen in third parties, or in the case of structural focus there is an unspecific reinforcement of structural themes. |  |  |  |
| Level 2  Foci acknowledge and exploration | The focus is suggested explicitly, either by the patient or the therapist exclusively. The other dyad member acknowledges it but does not work on it. The other member of the dyad is able to recognize it but there is no work on it.  For example, even though the therapist’s discourse goes around the foci, the patient is only able to say, “Yes, yes, it can be like that.” |  |  |  |
| Level 3  Work on the foci | Patient and therapist refer to foci, and their discourse goes around it; clearly both are working on it. |  |  |  |
