# Supplementary material for: Tracking Psychodynamic Foci: Trajectories Through the Therapeutic Process
Source: Front Psychol. 2022 Jun 6;13:786240. doi: 10.3389/fpsyg.2022.786240 (PMC9207400; doi:10.3389/fpsyg.2022.786240)
Supplement: Supplementary file 2 [file Table_2.DOCX]

### **Annex 2**

### Hierarchy of generic change indicators (listed in ascending order; Krause et al., 2007)

| **Level** | **Indicators** |
| --- | --- |
| I. Initial consolidation of the structure of the therapeutic relationship | 1. Acceptance of the existence of a problem.  2. Acceptance of his/her limits and of the need for help.  3. Acceptance of the therapist as a competent professional.  4. Expression of hope.  5. Questioning of habitual understanding, behavior and emotions.  6. Expression of the need for change.  7. Recognition of his/her own participation in the problems. |
| II. Increase in permeability toward new understandings | 8. Discovery of new aspects of self.  9. Manifestations of new behaviors and emotions.  10. Appearance of feeling of competence.  11. Establishment of new connections.  12. Reconceptualization of problems and/or symptoms.  13. Transformation of valorizations and emotions in relation to self or others. |
| III. Construction and consolidation of a new understanding | 14. Creation of subjective construct of self through the interconnection of personal aspects and aspects of the surroundings, including problems and symptoms.  15. Founding of the subjective constructs in own biography.  16. Autonomous comprehension and use of the context of psychological meaning.  17. Acknowledgment of help received.  18. Decreased asymmetry between patient and therapist.  19. Constructions of a biographically grounded subjective theory of self and others and of the relationship with surroundings. |

*Note*. Taken from Altimir et al. (2010)
